# A (3D-Nuclear) Space Odyssey: Making Sense of Hi-C Maps

**DOI:** 10.3390/genes10060415

**Published:** 2019-05-29

**Authors:** Irene Mota-Gómez, Darío G. Lupiáñez

**Affiliations:** Epigenetics and Sex Development Group, Berlin Institute for Medical Systems Biology, Max-Delbrück Center for Molecular Medicine, 10115 Berlin, Germany; Irene.MotaGomez-Argente@mdc-berlin.de

**Keywords:** 3D-chromatin organization, long-range gene regulation, chromosome conformation capture

## Abstract

Three-dimensional (3D)-chromatin organization is critical for proper enhancer-promoter communication and, therefore, for a precise execution of the transcriptional programs governing cellular processes. The emergence of Chromosome Conformation Capture (3C) methods, in particular Hi-C, has allowed the investigation of chromatin interactions on a genome-wide scale, revealing the existence of overlapping molecular mechanisms that we are just starting to decipher. Therefore, disentangling Hi-C signal into these individual components is essential to provide meaningful biological data interpretation. Here, we discuss emerging views on the molecular forces shaping the genome in 3D, with a focus on their respective contributions and interdependence. We discuss Hi-C data at both population and single-cell levels, thus providing criteria to interpret genomic function in the 3D-nuclear space.

## 1. Introduction

The three-dimensional (3D) spatial organization of chromatin within the nucleus represents a crucial step that bridges the linear information encoded in genomes with its capacity to execute complex but precise transcriptional programs. Therefore, chromatin organization underlies the molecular basis of a wide range of biological processes such as cell cycle control and DNA replication, repair, or maintenance [1]. By physically connecting distant cis-regulatory elements with gene promoters, through chromatin folding, transcriptional responses are executed with extraordinary precision in both space and time [2]. Furthermore, the capacity of chromatin to mediate long-range interactions has been proposed to underlie the regulatory expansion of vertebrate genomes and the exponential increase of pleiotropic gene functions that are central to develop complex organisms [3]. Compelling evidence accumulates, supporting the important role of spatial organization in development and delineating its alterations as prominent causes of human diseases, such as congenital malformation and cancer [4,5], or evolutionary adaptation [6]. Therefore, the elucidation of the molecular mechanisms controlling 3D-chromatin organization represents one of the cornerstones of modern biology.

For decades, the scientific community has been intrigued by the nature of the physical interactions between regulatory elements and genes, prompting the development of novel analytical and experimental strategies for their investigation. While microscopy-based approaches, such as Fluorescent In-Situ Hybridization (FISH), successfully settled the mechanistic basis of these functional contacts [7], it was not until the recent development of Chromosome Conformation Capture (3C) methods, that chromatin interactions could be investigated on a genomic scale [8]. The most informative of these technologies, Hi-C, relies on a combination of DNA proximity ligation together with high-throughput sequencing that allows the examination of all interacting loci across the genome, thus providing an overview of the nuclear organization of chromatin [9].

Almost 10 years after the release of the first Hi-C maps, the field has expanded rapidly, in part due to the continuous improvement and decreasing costs of Next Generation Sequencing technologies. These advances led to the identification of chromatin interactions at an unprecedented resolution, yielding a massive amount of data, on a wide range of cells, tissues, and organisms. Yet, translating the signal of Hi-C maps into a meaningful biological output remains one of the biggest challenges of the field. It has become increasingly evident that Hi-C maps are the results of multiple, overlapping mechanisms, with a highly dynamic nature, that converge in a temporal “snapshot” of nuclear interactions. The individual dissection of these mechanisms is central to understand the underlying biology of such datasets.

In this review, we discuss current knowledge about the biology of chromatin interactions, to provide a framework that enables the interpretation of Hi-C maps. We evaluate fundamental mechanisms under the prism of the population-based nature of the technology, but contextualizing them with recent single-cell and super-resolution microscopy advances. The appropriate interpretation of chromatin interaction datasets will allow the navigation through a 3D-nuclear space, thus unlocking the true potential of these approaches to facilitate the interpretation and prediction of genomic function in biological processes.

## 2. Prominent Structures and Mechanisms of 3D Nuclear Organization

### 2.1. Transcription-Based Compartments

**Figure 1:**
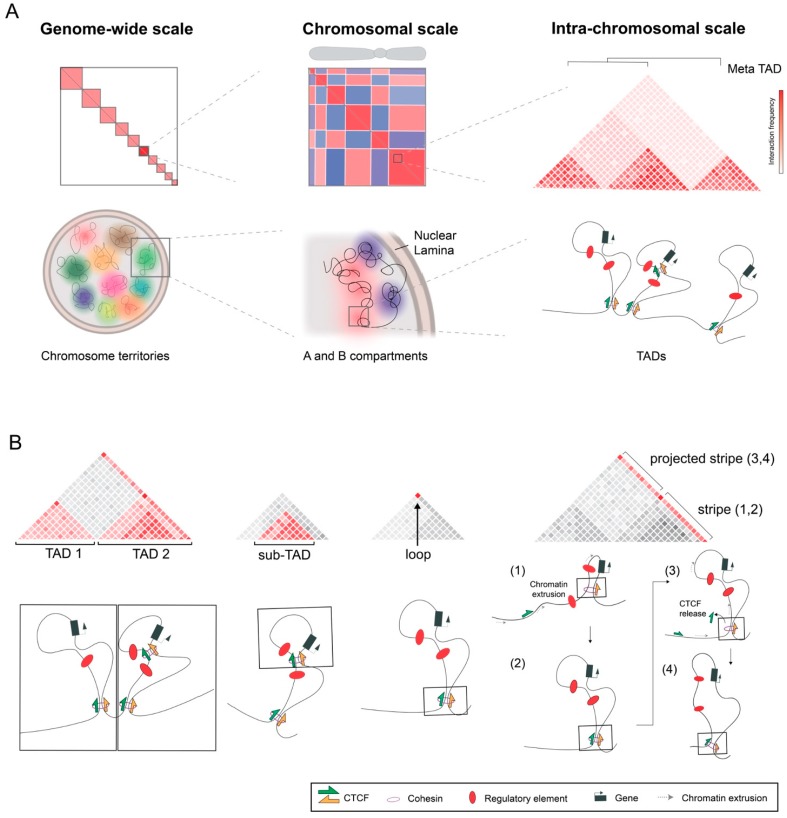
Prominent features observed in Hi-C maps. (**A**) At a genome wide level, chromosomes are organized according to distinct territories that are reflected by the degree on intra vs. inter-chromosomal interactions. At the chromosomal level, distinct regions are organized into multi-megabase A/B compartments (red/blue) according to transcriptional and epigenetic status. Within compartments, chromatin is organized into TADs (average size of 880 kb) that also show a nested hierarchy into meta-TADs. (**B**) At the TAD level, multiple features can be observed, such as sub-TADs (average size of 185 kb), loops or stripes/tracks. Squared regions represent the genomic region in the chromatin fiber where chromatin interactions occur and that originates the observed feature. Note, the dynamic nature of extrusion that is associated to the formation of stripes/tracks, where the structures can be extended upon release of individual CCCTC-binding factor (CTCF) molecules from chromatin.

As highlighted previously, 3C-based technologies and, in particular, Hi-C represented a novel approach to interrogate chromatin interactions in a high-throughput manner. With this technology, large genome-wide matrices of interaction could be reconstructed and visualized, with squares displaying interaction frequencies, depicted by color intensity, between pairs of loci on the linear genome. Early Hi-C maps profiling mammalian cells were generated at 1 Megabase (Mb) resolution, each bin containing interactions between fragments within a 1 Mb window [9]. The analysis of these maps supported findings from the 80s [10], confirming the organization of individual chromosomes in distinct nuclear territories, reflected by an overall higher degree of intra- vs. inter-chromosomal interactions (Figure 1A).

Those early findings, based on FISH and chromosome painting techniques, also delineated a prominent separation between euchromatic and heterochromatic regions. This phenomenon is also observed in Hi-C maps, depicted by a plaid pattern of enriched or depleted interactions within and between chromosomes [9]. These segregated regions were denominated A/B compartments, covering genomic extensions of several Mb. Through the intersection with expression and chromatin accessibility datasets, it became clear that A compartments overlapped with open, transcriptionally-active regions, whereas B compartments corresponded to regions with closed, inactive chromatin. This correlation also reflects the cell-type specific and dynamic nature of A/B compartments. For instance, a significant reconfiguration of these units is observed during differentiation, with noticeable “compartmental switching” events and a reinforcement of B-type interactions that correlate extensively with gene expression changes [11,12,13,14].

To date, it is not entirely clear how the segregation of these compartments occurs, but interaction data at high resolution revealed compartmental associations that correlate well with the existence of specialized sites within the nucleus, such as Polycomb-repressed, Lamina-Associated Domains (LADs), or Nucleolus-Associated Domains (NADs) [14]. Furthermore, novel theories suggest an involvement of phase-separation mechanisms on the segregation into A/B compartments [15].

### 2.2. Domains and Loops Formed by Chromatin Extrusion

With further improvements on Hi-C resolution (20–40 kilobases, kb), an additional level of chromatin folding was revealed in mammalian genomes, at the sub-megabase scale. These units were denominated Topologically Associated Domains (TADs), defined as genomic regions with increased contact frequency and largely insulated from other neighboring regions [16,17] (Figure 1A,B). Compared to compartments, TADs are shorter, with an average size around 880 kb in human and mouse genomes. As such, TADs usually lie within A or B compartments, representing regulatory units that can be either active or inactive in a certain cell type. TADs are delineated by boundary regions that are enriched in housekeeping genes or tRNAs but, most notably, in the CCCTC-binding factor (CTCF) [16]. The position of TADs and boundary regions is remarkably stable between cell types or tissues, even displaying evolutionary conservation among species [12,16]. The disruption of TADs is a prominent mechanism of human disease, leading to aberrant gene expression and causing congenital disease or cancer [18,19,20,21,22]. Their striking overlap with domains with a regulatory potential [23], delineates TADs as a fundamental functional unit during development that enables proper enhancer-promoter communication.

TADs are closely related to another prominent feature of Hi-C maps: chromatin loops (Figure 1B). Loops are observed as focal points of interaction with a medium size of 185 kb and that tend to appear at upper corners of TADs, although they can also occur within TADs [14]. A large fraction of chromatin loops (86%) are bound by the cohesin subunits RAD21 and SMC3, as well as by CTCF, this last one usually displaying convergent orientation on its DNA-binding motif between loop-anchor points [14]. Remarkably, the inversion of individual CTCF sites has an impact on the formation of loops, with the potential of redirecting interactions [24,25]. At TAD boundaries, clusters of CTCF-binding sites in divergent orientation are generally found, serving as anchor points for interactions that project in opposite directions and provide an insulator function [14,26,27]. In contrast to CTCF-associated loops, which tend to be conserved between cell types and during differentiation, CTCF-independent loops display a more dynamic nature and associate to transcriptional interactions, such as enhancer-promoter or Polycomb-mediated contacts ([11,14]; preprints: [28,29]).

The most prominent theory by which TADs and chromatin loops are formed is the loop-extrusion model [30,31]. In this model, loop-extruding factors (LEF) extrude chromatin until this activity is blocked by boundary elements (BEs). The extrusion process would therefore allow the physical contact of the loci delimited by BEs while, at the same time, constrain the interactions beyond these points. The cohesin complex, with its tripartite ring structure, was proposed as the main LEF during interphase, delineating a highly dynamic process given the nature of the complex and its transient binding to chromatin. CTCF itself was proposed as the BE factor, due to its prominent association with TAD features and the consistent results observed in experimental perturbations affecting individual binding sites [24,25,31,32]. Therefore, loop extrusion provides a model that integrates the dynamics of chromatin with the spatial structure that is visible in interaction maps.

### 2.3. Opposing Forces Organizing the 3D Chromatin Space

The identification of potential key players in 3D-chromatin organization, such as CTCF, cohesin or transcription, led to a plethora of genetic studies to elucidate their individual role. These experiments provided important information to understand the interdependence of the aforementioned mechanisms and how they are reflected in Hi-C maps.

The acute depletion of CTCF in mouse cells, via induced-auxin degron, resulted in a loss of loop domains [33,34]. However, CTCF restoration resulted in a rapid re-structuration of chromatin with the emergence of TADs [33]. Interestingly, loss of CTCF did not disturb higher-order chromatin structures, such as A/B compartments, suggesting an independent action of compartmentalization and loop-extrusion mechanisms. Similar conclusions were observed upon depletion of the cohesin subunit NIPBL, responsible for loading cohesin to chromatin. In this case, a loss of TADs and loops was found, whereas A/B segregation not only persisted, but also shorter compartments emerged [35]. This finer level of compartmentalization correlated better with transcriptional activity and epigenetic marks when compared to wildtype Hi-C maps. The depletion of RAD21, another core component of the cohesin complex, also resulted in a global disappearance of loops and TADs but and a general reinforcement of compartments [34,36]. Overall, these experimental approaches led to the conclusion that chromatin loop extrusion represents an important layer of organization that is imposed on an already preformed segregation into active and inactive compartments [37].

Studies depleting the cohesin-releasing factor WAPL increase the persistence of the cohesin complex on chromatin, thus providing important insights on the nature of the loop extrusion process [34,38,39]. These studies not only demonstrated a stabilization on existing loops, but also the formation of new ones connecting larger genomic distances. These results depict loop extrusion as a dynamic process, highly dependent on the residence time of both CTCF and the cohesin complex on chromatin. While lowering the residence time of cohesin decreases loop strength, an increase has the opposite effect, allowing the complex to overcome boundary elements and thus extrude larger chromatin sections that generate new loop structures.

The extension of chromatin-interaction studies to other organisms also provided essential clues to understand the relation between compartment segregation and chromatin extrusion. Among vertebrates, loop extrusion, and the CTCF orientation code appear as conserved mechanisms of chromatin organization [26,40,41], although those rules are not universal across the entire animal kingdom. This is a remarkable observation, mainly due to the evolutionary conservation of CTCF across metazoans and the striking similarities of its DNA-binding motif between species. Most of the experimental evidence of such discrepancy comes from studies in *Drosophila melanogaster*, where initial low-resolution Hi-C maps also revealed the existence of TAD structures around the size of 100 kb [42], with boundaries frequently bound by insulator proteins, such as BEAF-32, Chromator or CP190, but not by divergently-oriented CTCF clusters [43]. High-resolution Hi-C maps revealed the existence of a reduced number of chromatin loops, which were frequently associated to the Polycomb complex, as well as a finer division of TAD into smaller domains in the range of 10 kb that were subsequently denominated as compartmental domains [44,45]. Further analyses highlighted that chromatin organization in *Drosophila* is better explained by compartmentalization based in transcriptional status, with a minor influence of insulator proteins [44]. While in mammals CTCF is crucial for viability [46], in flies it is largely dispensable for embryonic development [47]. Therefore, it seems that, at least in *Drosophila*, TAD formation by loop extrusion does not plays a prominent role in chromatin organization [37]. Such findings are consistent with studies in other organisms where CTCF homologous cannot be found, such as yeast, bacteria, or plants, and where a remarkable level of organization into TAD-like domains can be noted [48,49,50]. In these organisms, transcription-based compartmentalization has also been proposed as the main force organizing chromatin [37]. It is also important to note that X chromosomes of *Caenorhabditis elegans* display structures that are reminiscent of TADs and that have been associated with another extruding complex, condensin, as well as with the dosage compensation complex (DCC) [51]. These findings suggest the potential existence of additional mechanisms that also contribute to shape chromatin within the nucleus.

Finally, the role of transcription in establishing 3D-chromatin organization has been also thoroughly investigated. Experiments in *Drosophila* showed that at early phases of development, when transcription is limited, chromatin is largely unorganized [52]. However, the inhibition of transcriptional elongation has a small effect on chromatin organization, thus being largely dispensable for domain formation [52]. In contrast, the inhibition of transcriptional initiation or Pol II recruitment to chromatin displays a more dramatic effect, reducing TAD insulation strength and increasing inter-TAD interactions [44,53]. In mammals, A/B compartmentalization can be prominently observed in mouse sperm, which is transcriptionally inactive [54,55,56]. However, CTCF-independent TAD boundaries form at the promoters of genes activated during neuronal differentiation [11]. Overall, these studies suggest that, although active transcription is likely not essential for compartmentalization into regulatory domains, certain components of the transcriptional machinery itself might play a role in the process.

In summary, two opposing mechanisms, a transcription-based that segregates chromatin into compartments according to epigenetics states, and a superimposed cohesin-dependent mechanism that generates TADs and loop-structures, act together to organize the genome of most vertebrate species. Outside this clade, transcriptional compartmentalization appears as the major contributor organizing chromatin in the 3D-nuclear space. Studies in additional species and further functional validations will help to infer the degree of evolutionary conservation of these two mechanisms and to better elucidate their functional relation, both important aspects to understand their individual contribution to Hi-C maps.

## 3. Additional Features of Hi-C Maps

Besides the prominent features described in the previous section, Hi-C maps also display additional structures that are linked to the explained mechanisms. In this section, we provide an overview of these features and the forces shaping them.

### 3.1. Hierarchies between and within TADs

In general, TADs represent a stable and largely invariant scaffold of genomic organization across different cells/tissues and during development. Nevertheless, additional levels of organization can be observed between and within TADs, in some cases displaying a marked cell-to-cell variability. One prominent example are meta-TADs, large spatial structures that connect groups of linearly-consecutive TADs [13] (Figure 1A). These units were identified in mouse embryonic stem cells, undergoing profound reorganization during differentiation into the neuronal lineage *in vitro*. Meta-TADs reflect a nested hierarchy of TADs within A/B compartments, thus providing a link between the molecular mechanisms of both features. In a recent study, TAD cliques were identified, which represent associations between non-linear, B-compartment TADs that are prominently reinforced during differentiation according to changes in chromatin compaction and gene repression [57].

Several studies have highlighted the further subdivision of TADs into smaller and nested structures such as sub-TADs, insulating neighborhoods or CTCF-contact domains (CCDs) [14,58,59,60,61] (Figure 1B). With a medium size on 185 kb in mammalian genomes, these internal structures display similar characteristics compared to TADs, in regard to increased self-interactions and the association with CTCF/cohesin-mediated loops. However, they also differ notably on their cell-specific nature and especially on their degree of insulation, allowing significant interactions with neighboring regions. In addition, high-resolution Hi-C maps (0.75–1 kb) showed that mammalian TADs can also be subdivided into finer compartments related to transcriptional activity, which are largely independent of CTCF binding and usually delimited by the promoters of genes with high levels of transcription [11,14]. Recent studies applied Micro-C, a variant of Hi-C that uses micrococcal nuclease (MNase) to fragment chromatin and to achieve better resolution at lower fragments, for the investigation of mammalian chromatin organization at nucleosome resolution (preprints: [28,29]). Those studies also confirmed the existence of transcription-related sub-TAD structures on the range of 5–10 kb and denominated micro-TADs. This phenomenon is reminiscent of the finer domains observed in *Drosophila* [44,45], highlighting again the overlap between compartmentalization and extruding forces that emerge from mammalian Hi-C maps.

### 3.2. Stripes or Tracks

Another important feature of Hi-C maps are stripes or tracks, which reflect large segments of interaction between a certain locus and a contiguous region of the genome (Figure 1B). A recent study revealed that stripes are generally associate to the edges of TADs, with stripe anchors coinciding with loop-anchor points [62]. At domains, stripes can appear symmetrically or asymmetrically, an effect that seems to be influenced by clustering of CTCF sites at one or both sides. In some cases, stripes can be prolonged beyond a CTCF-mediated loop (Figure 1B), upon dissociation of individual CTCF molecules from chromatin. These observations agree with the loop-extrusion model, in which the formation of stripes is predicted [30]. Furthermore, stripe formation can display prominent tissue-specific differences and have an impact on gene expression and the appearance of certain phenotypes [63]. From a mechanistic perspective, stripes represent the process of scanning along the chromatin fiber by the cohesin complex. Since Hi-C maps represent static “snapshots” of cell populations and the process of extrusion is dynamic, in individual cells cohesin can be located at different positions while extruding, which on average will be observed as a continuous track.

### 3.3. Assembly-Related Signatures

A proper interpretation of a Hi-C signal relies on mapping the data against a genomic assembly that matches the profiled sample. When a discrepancy exists, specific signatures arise that, in most cases, can be identified by genome-wide analyses or even manual inspection. This property has been effectively exploited to expand the utilization of Hi-C methods beyond the study of chromatin interactions.

One of these applications is the application of Hi-C as a tool to identify structural variations associated to human disease. This strategy takes advantage of the fact that genomic rearrangements generally alter the distance between genomic loci, causing signal discrepancies due to data alignment against a reference genome that does not contain the variation. Depending on the specific pattern observed in the Hi-C map, deletions, inversions, duplications or translocations can be identified upon visual inspection (reviewed in [4]). The identification of these signatures can be applied on large cohort screens or to reconstruct the mutational history of complex rearrangements like chromothripsis [64].

Another prominent application of Hi-C relates to its utilization to scaffold highly-fragmented genomic assemblies into entire chromosomes [65] (preprint: [66]). Through a probabilistic model, genomic contigs are oriented and placed in appropriate linear order, according to their frequency of interactions from Hi-C data. This strategy is particularly suitable for non-model organisms where the access to genomic data is limited. While these assemblies might contain numerous gaps between the assembled contigs, due to repetitive sequences or low coverage, they facilitate a broad overview of 3D-chromatin organization.

## 4. Bridging the Gap: From Population-Averaged to Single-Cell Interactions

The study of chromatin interactions bears an important limitation—the information that can be retrieved from an individual locus is limited by the number of copies present in the genome, two in general for diploid cells. 3C-based technologies have circumvented this problem by profiling interactions of millions of pooled cells, to produce statistically significant results. An important unresolved issue related to this strategy is whether TADs, as observed in Hi-C maps, represent stable 3D ensembles or rather an average of distinct conformations derived from the large number of profiled cells. Polymer model simulations from bulk 5C data, suggested the second scenario, with multiple 3D configurations constrained by the presence of TAD boundary elements [67]. Recent advances in single-cell approaches and high-throughput microscopy provided novel tools that are facilitating the elucidation of this issue and a more comprehensive study of chromatin interactions at an individual cellular level.

The first single-cell Hi-C study revealed that maps of pooled single-cell matrices had invariable domains that corresponded to those seen in the bulk Hi-C maps, despite the limited information retrieved from individual cells [68]. However, more recent single-cell Hi-C protocols have improved map resolution, providing better insights on the individual variation between cells [69,70,71]. In general, A/B compartments are mostly invariable at the single-cell level. However, TAD conformations in single cells display a high degree of variability, ranging from compacted to more extended structures [70]. These results are consistent with polymer simulations [67], and the loop extrusion model [30]. In general, single-cell data reflects the dynamic process of TAD formation, with boundary regions that, despite not providing complete insulation in some cells, overall constrain interactions on an averaged population.

Super-resolution microscopy has been also employed to interrogate 3D-chromatin organization at a single-cell level. For example, by combining Oligopaint probes and Stochastic Optical Reconstruction Microscopy (STORM) it was possible to visualize a 1.2 Mb region in mammalian cells with nanometer-scale precision [72]. This method confirmed the high-degree of cell-to cell variability also noted by single-cell Hi-C methods. In individual cells, TAD boundaries could be detected at any genomic position, but a strong preference was observed for CTCF/cohesin associated regions. Upon cohesin removal, single-cells still retain the capacity to form TAD-like structures, except that boundary regions did not match with CTCF-binding domains but were randomly distributed, thus explaining the loss of TAD structures in bulk maps [34,35]. These results suggest that cohesin is not required for maintenance of chromatin folding, but rather to position boundaries at CTCF-binding sites [72]. Similar approaches have been also been employed to visualize genomic folding in *Drosophila*, revealing a high degree of cell-to-cell heterogeneity and the dynamic nature of TAD formation. Nevertheless, in this species, TADs also appear to be largely based on the segregation between active and inactive chromatin domains [73,74].

In summary, the application of these novel approaches, to profile 3D-chromatin organization, provides results at the individual cellular level that recapitulates observations obtained from bulk data. Therefore, TADs can adopt multiple configurations, with higher probabilities to match the structures observed in averaged cells, but where insulation and boundary positioning do not appear as absolute values.

## 5. Outlook

Hi-C datasets represent a powerful tool to understand how chromatin is organized within the nucleus to effectively exert its biological function. Notably, chromatin organization is reflected at multiple genomic scales: from specific chromosomal territories to functional loops that connect enhancers to their cognate promoters. Therefore, distinct molecular forces impose constrains at these levels, thus converging on the complex interaction patterns that are visualized in Hi-C maps.

Great advances have been made towards the identification of the essential factors and mechanisms that organize the genome within the nuclear space. Among them, transcription and loop extrusion by CTCF/cohesin appear as prominent players, acting as opposing forces that lie in a delicate balance. However, interspecies comparisons, such as between flies and mouse or human, reveal an absence of universal rules of chromatin organization. While compartmentalization appears to be an ancient feature of 3D-chromatin organization, domain formation by loop extrusion seems to be a more recent evolutionary acquisition. This type of organization can impose over transcription-based compartmentalization, inducing additional spatial constrains in the form of boundary elements. Profiling chromatin interactions in additional species along the phylogenetic tree will not only help to understand the origin and functional implications of this innovation, but also to the potential identification of other factors with the capacity of shaping the genome in 3D.

Novel methods, such as single-cell or super-resolution microscopy, are helping to fill the gap between average population and the events occurring at a cellular level. As a field in development, it is expected that these technologies will acquire better resolution and throughput capacity. This will enable profiling chromatin interactions on vast amounts of individual cells from tissues and organs, thus providing exciting insights on how average conformations are shaped to meet specific transcriptional requirements in time and space. Applying this knowledge to identify subtle changes in interaction maps and their underlying causes and effects represents the next frontier in the field. Such knowledge would have important implications for the identification of novel mechanisms underlying human disease or evolutionary traits, eventually leading to better predictive models that might improve the connection between visual patterns of 3D-chromatin interaction and biological processes.
